# STransfer: a transfer learning-enhanced graph convolutional network for clustering spatial transcriptomics data

**DOI:** 10.1093/bioinformatics/btag049

**Published:** 2026-01-27

**Authors:** Chaojie Wang, Xin Yu

**Affiliations:** School of Mathematical Sciences, Jiangsu University, Zhenjiang, Jiangsu 212013, China; School of Finance & Economics, Jiangsu University, Zhenjiang, Jiangsu 212013, China

## Abstract

**Motivation:**

Capturing spatial structure is fundamental to the analysis of spatial transcriptomics data. However, most existing methods focus on clustering within individual tissue slices and often ignore the high inter-slice similarity inherent in multi-slice datasets.

**Results:**

To address this limitation, we propose STransfer, a novel transfer learning framework that combines graph convolutional networks (GCNs) with positive pointwise mutual information (PPMI) to model both local and global spatial dependencies. An attention-based module is introduced to fuse features from multiple graphs into unified node representations, facilitating the learning of low-dimensional embeddings that jointly encode gene expression and spatial context. By transferring knowledge from labeled slices to adjacent unlabeled ones, STransfer significantly enhances clustering accuracy while reducing manual annotation costs. Extensive experiments demonstrate that STransfer consistently outperforms state-of-the-art methods in both spatial modeling and cross-slice transfer performance.

**Availability and implementation:**

The code for STransfer has been uploaded to GitHub: https://github.com/Saki-JSU/Publications/tree/main/STransfer.

## 1 Introduction

Spatial transcriptomics (ST) has emerged as a transformative technology that enables high-throughput gene expression profiling while preserving the spatial context of tissue architecture (Ståhl *et al.* 2016, Zeng *et al.* 2023). By simultaneously capturing gene expression patterns and their spatial localization, ST provides unprecedented insights into tissue heterogeneity, cell–cell interactions, and the spatial organization of biological processes (Williams *et al.* 2022). One of the fundamental computational tasks in ST analysis is unsupervised clustering, which aims to identify spatially coherent and transcriptionally distinct regions (Teng *et al.* 2022). Accurate clustering is crucial for downstream tasks such as tissue annotation (Rao *et al.* 2021), multimodal analysis (Min *et al.* 2024, Niu *et al.* 2024, Xue *et al.* 2024, Zhao and Min 2025), deconvolution inference (Huang *et al.* 2025), and disease diagnosis (Chen *et al.* 2023, Shi *et al.* 2023).

A variety of computational methods have been developed to enhance clustering in spatial transcriptomics by incorporating spatial information. Among classical statistical approaches, Seurat (Hao *et al.* 2024) is a widely used toolkit that performs clustering by applying the Louvain algorithm to a low-dimensional representation of the data obtained via principal component analysis (PCA). In addition, Dries *et al.* (2021) employed a hidden Markov random field (HMRF) model to identify spatial domains by modeling the joint probability of each cell’s intrinsic and extrinsic states. Zhao *et al.* (2021) proposed BayesSpace, a fully Bayesian approach that leverages spatial neighborhood information for resolution enhancement and clustering of ST data.

Recently, numerous methods have adopted graph-based deep learning models, particularly graph convolutional networks (GCNs) (Scarselli *et al.* 2009), to integrate gene expression and spatial context (Sun *et al.* 2024). SpaGCN by Hu *et al.* (2021) was one of the first to apply GCNs in ST, jointly modeling gene expression, spatial coordinates, and histology. By aggregating gene expression from neighboring spots, SpaGCN effectively identifies spatial domains with coherent transcriptomic and histological features. Building on this foundation, Li *et al.* (2022) introduced an unsupervised GCN-based clustering method to improve cell-type discovery, while Xu *et al.* (2022) developed DeepST, which combines a graph neural network (GNN) autoencoder with a denoising autoencoder to learn latent representations from augmented ST data. They also integrated data across batches using domain adversarial networks. Brbić *et al.* (2022) presented STELLAR, a supervised geometric deep learning method for cell-type discovery and identification in spatially resolved single-cell datasets. Long *et al.* (2023) proposed GraphST, a self-supervised contrastive learning framework for spatial clustering, multi-sample integration, and cell-type deconvolution. More recently, Xu *et al.* (2024) presented SEDR, which employs a deep autoencoder with masked self-supervised learning to derive low-dimensional gene expression embeddings, further integrated with spatial information via a variational graph autoencoder. To address the instability of graph-based models caused by the high sparsity of ST data, Min *et al.* (2025) proposed SpaMask, which enhances model robustness through a dual-masking graph autoencoder combined with contrastive learning. In addition, Niu *et al.* (2025b) introduced SpatialCVGAE, which further enhances model stability and robustness by integrating multi-dimensional high-variable gene expressions with multiple spatial graphs.

Despite their effectiveness, the majority of existing methods operate on individual tissue slices, treating each as an independent unit (Wang *et al.* 2025). However, in many experimental settings, multiple adjacent tissue sections are collected from the same sample or biological system, and these multi-slice datasets inherently exhibit strong inter-slice similarity in both spatial patterns and transcriptional features (Li and Zhou 2022). Failing to exploit this cross-slice redundancy leads to suboptimal clustering performance and limited generalizability, especially in slices with noisy or missing labels. To address these limitations, recent studies have begun to explore multi-slice modeling strategies. For example, STG3Net (Fang *et al.* 2024) and SpaBatch (Niu *et al.* 2025a) were proposed to correct batch effects across multiple slices. More recently, Fang and Min (2025) introduced SpaCross, which employs a cross-masked graph autoencoder to reconstruct gene expression features while preserving spatial relationships and mitigating identity mapping issues. By jointly balancing local spatial continuity and global semantic consistency, SpaCross improves spatial pattern recognition and cross-slice integration.

In this paper, we introduce STransfer, a novel transfer learning framework specifically designed for clustering multi-slice spatial transcriptomics data. STransfer employs a dual-graph strategy to model both local spatial relationships using standard GCNs and global statistical dependencies through positive pointwise mutual information (PPMI)-based adjacency matrices (Wu *et al.* 2020). This design enables the model to effectively capture fine-grained spatial interactions as well as long-range co-expression patterns. To further enhance representation learning, we incorporate an attention-based feature fusion module that integrates signals from multiple graph structures into unified node embeddings. These low-dimensional embeddings jointly encode spatial context and gene expression, serving as robust inputs for clustering.

A distinctive feature of STransfer is its cross-slice transfer capability. By learning transferable knowledge from labeled slices, the model can generalize to adjacent unlabeled slices, thereby improving clustering performance without additional manual annotations. This is particularly valuable in practical scenarios where exhaustive labeling is labor-intensive, time-consuming, or biologically infeasible. To achieve this, STransfer adopts a transfer learning strategy that leverages both generative and adversarial mechanisms (Tzeng *et al.* 2017).

Specifically, the generative module synthesizes pseudo-samples in the target (unlabeled) slice domain that mimic the distribution of real data from the source (labeled) domain. At the same time, a domain discriminator is trained to distinguish between real and generated samples. Through adversarial training, the generator learns to produce increasingly realistic pseudo-samples that confuse the discriminator, ultimately making it difficult to distinguish between source and target domains. This adversarial interplay facilitates domain alignment, enabling the effective transfer of knowledge from labeled slices to unlabeled ones while preserving biological structure and consistency.

We validate the effectiveness of STransfer through extensive experiments on several benchmark spatial transcriptomics datasets. Results demonstrate that our method consistently outperforms existing approaches in terms of clustering accuracy, spatial coherence, and transferability across tissue slices.

This paper is organized as follows: Section 2 describes the datasets used, details the structure of STransfer, and outlines the training process. Section 3 presents the performance of models in various datasets. Section “Discussion” further discusses the results.

## 2 Materials and methods

### 2.1 Datasets

**Figure 1:**
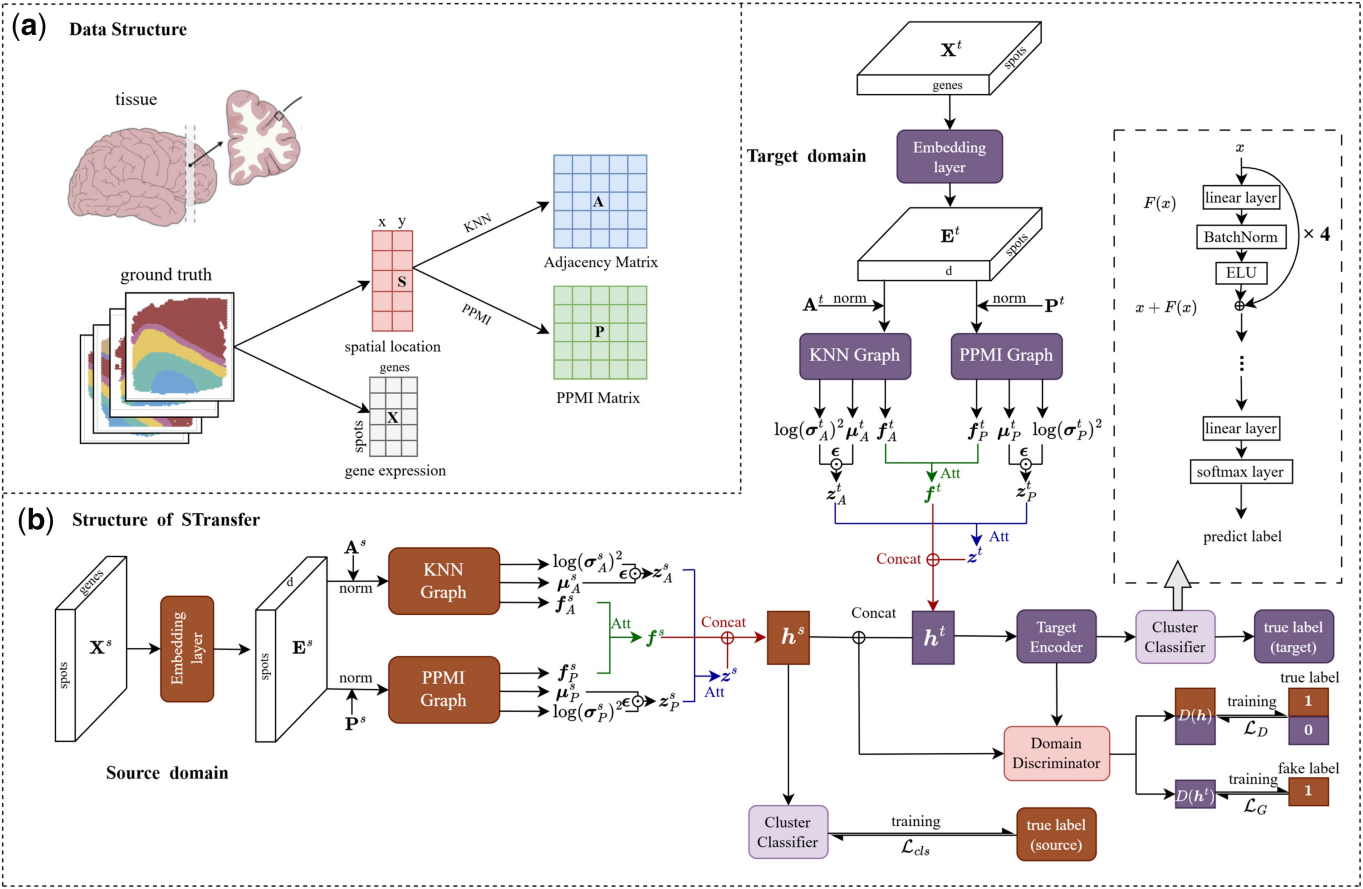
Overview of STransfer. (a) the introduction of ST data and the process of graph construction from spatial coordinate matrix. (b) The architecture of STransfer is composed of three major components: dual-graph convolution, cluster classifier, and domain adaptation module.

Spatial transcriptomics data typically consists of two key components: a gene expression matrix for spots and corresponding spatial coordinates. Each spot represents a small, localized area on the tissue slice, capturing the aggregated expression levels of thousands of genes. Formally, for each tissue slice, the data can be represented as a pair (X,S), where $X\in R^{N\times G}$ denotes the gene expression matrix with *N* spots and *G* genes, and $S\in R^{N\times 2}$ stores the two-dimensional spatial coordinates (e.g., x-y positions) of the spots on the tissue. Figure 1a briefly introduces the structure of ST data.

To evaluate the performance and generalizability of our method, this paper benchmarks STransfer on five publicly available and widely used spatial transcriptomics datasets:

**DLPFC** (Maynard *et al.* 2021): This dataset comprises 12 human dorsolateral prefrontal cortex (DLPFC) slices generated using the 10x Genomics Visium platform, accessible via the spatialLIBD project (Pardo *et al.* 2022). Each slice contains between 3460 and 4789 spatial spots, profiling 33 538 genes. Manual annotations segment each slice into five to seven anatomically distinct regions, including cortical layers and white matter.

**HPR** (Moffitt *et al.* 2018): This dataset includes five annotated slices from the mouse hypothalamic preoptic region (HPR), generated using a combination of single-cell RNA sequencing and the MERFISH (multiplexed error-robust fluorescence in situ hybridization) technique (Chen *et al.* 2015). Each slice contains between 5488 and 5926 spots and covers expression profiles for 155 genes. The slices are manually annotated into eight distinct anatomical regions.**mPFC** (Wang *et al.* 2018): This dataset consists of three annotated slices from the mouse medial prefrontal cortex (mPFC), generated using the STARmap (spatially resolved transcript amplicon readout mapping) technique. Each slice comprises 1049 to 1088 spatial spots with gene expression measurements for 166 genes. Manual annotations divide each sample into four anatomically defined regions.

**CRC** (Valdeolivas *et al.* 2024): This dataset comprises a pair of slices from a colorectal cancer (CRC) patient, serving as technical replicates generated using the 10x Genomics Visium platform. The two sections contain 1691 and 2128 spots, respectively, with each spot profiling 36 601 genes. Manual annotation further partitioned each sample into five distinct tissue regions.

**OC** (Ren *et al.* 2025): This dataset consists of four human ovarian cancer (OC) tissue samples obtained from the same patient and profiled using four spatial transcriptomics platforms: CosMx, Xenium, Stereo-seq, and Visium HD. Each platform provides a single annotated tissue section, with 9999–10 000 spatial spots after downsampling to ensure comparable spot densities across technologies. Gene coverage varies by platform, including 6175 genes (CosMx), 5001 genes (Xenium), 32 008 genes (Stereo-seq), and 18 085 genes (Visium HD). Manual expert annotation further partitions each tissue slice into 13 tumor–microenvironment regions.

Table 1 presents a brief summary of the three datasets analyzed in this study.

**Table 1:** Overview of spatial transcriptomics datasets used for evaluation.

Datasets	SeqTech	Species	Slices	Spots	Genes	Regions
DLPFC	10x Visium	Human	12	3460–4789	33 538	5–7
HPR	MERFISH	Mouse	5	5488–5926	155	8
mPFC	STARmap	Mouse	3	1049–1088	166	4
CRC	10x Visium	Human	2	1691–2128	36 601	5
OC	CosMx	Human	1	10 000	6175	13
	Xenium	Human	1	9999	5001	13
	Stereo-seq	Human	1	10 000	32 008	13
	Visium HD	Human	1	9999	18 085	13

### 2.2 Graph construction

To effectively incorporate spatial information into downstream representation learning and clustering, we construct a graph G=(V,E) for each tissue slice, where each node $v_{i}\in V$ corresponds to a spot, and edges E encode spatial relationships among spots. The spatial structure is derived from the two-dimensional spatial coordinate matrix $S\in R^{N\times 2}$, where *N* is the number of spots in a slice. In this paper, we explore two types of adjacency matrix construction strategies.

K-Nearest Neighbors (KNN) adjacency is the most straightforward approach to building spatial graphs, which connects each spot to its *k* nearest neighbors in Euclidean space. For each node $v_{i}$, we compute pairwise distances using the spatial coordinates in **S**, and select the *k* closest nodes to form undirected edges. The resulting binary adjacency matrix $A={\left(a_{ij}\right)}_{N\times N}$ is defined as:


(1) $$a_{ij}=\{\begin{array}{c} 1,\;\text{if}\;v_{j}\in \text{KNN}(v_{i}),\\ 0,\;\text{otherwise}. \end{array}$$

This formulation captures local spatial connectivity, ensuring that each node aggregates information from its immediate neighborhood.

While the KNN graph encodes only local spatial proximity, it lacks the ability to model long-range statistical associations. To address this, we further refine the spatial graph using positive pointwise mutual information, inspired by techniques in word embedding and knowledge graph modeling (Levy and Goldberg 2014).

Starting from the KNN-based adjacency matrix **A**, we perform a random walk to obtain a transition probability matrix **P**. Specifically, we simulate truncated random walks over the graph and estimate co-occurrence probabilities between node pairs. Then, the PPMI matrix $P={\left(p_{ij}\right)}_{N\times N}$ is computed as:


(2) $$p_{ij}=\max\left(0,\;\text{log}\;\frac{P(i,j)}{P(i)P(j)}\right),$$

where P(i,j) is the joint probability of nodes $v_{i}$ and $v_{j}$ co-occurring in random walks, and P(i), P(j) are their marginal probabilities. This formulation highlights node pairs with strong co-occurrence patterns beyond local proximity, effectively capturing global spatial relationships in the tissue structure.

By combining both adjacency matrices, we enable the model to integrate multi-scale spatial dependencies—local through KNN, and global through PPMI-enhanced structural information. Figure 1a describes the process of graph construction from spatial coordinate matrix.

### 2.3 Model architecture

STransfer is designed as a transfer learning framework that enables knowledge transfer from annotated (source) tissue slices to unannotated (target) slices in spatial transcriptomics data. Its architecture is composed of three major components: *dual-graph convolution*, *cluster classifier*, and *domain adaptation module*. The model jointly leverages local and global spatial dependencies and aligns latent representations across domains to facilitate cross-slice generalization. Figure 1b presents the structure of STransfer.

Given a spatial transcriptomics dataset with multiple tissue slices, we treat each slice as an individual domain. For any pair of slices, we designate the labeled slice as the source domain and the unlabeled slice as the target domain. Formally, for the source domain (denoted as *s*), the input includes the gene expression matrix $X^{s}$ along with two graph structures: the KNN-based adjacency matrix $A^{s}$ and the PPMI-based adjacency matrix $P^{s}$. For the target domain (denoted as *t*), the corresponding matrices are $X^{t}$, $A^{t}$, and $P^{t}$, with no manual region labels provided. The goal of STransfer is to transfer clustering-relevant knowledge from the source domain to the target domain. This enables the model to generate accurate and biologically meaningful spatial clusters on the unlabeled target slice, without requiring any manual annotations.

### 2.4 Dual-graph convolution

To effectively capture both local and global spatial dependencies in spatial transcriptomics data, STransfer introduces a dual-graph convolution module comprising two parallel GCN branches: one based on the KNN graph **A**, and the other on the PPMI graph **P**.

Given the input gene expression matrix $X\in R^{N\times G}$, we first project the high-dimensional data into a lower-dimensional latent space using a linear embedding layer. The embedded feature matrix $E\in R^{N\times d}$ is then fed into two separate GCN pipelines: the KNN-graph branch leverages the adjacency matrix **A** to capture local spatial information, while the PPMI-graph branch uses **P** to incorporate global structural context. Each GCN branch consists of three graph convolutional layers with identical architecture but independent parameters. These layers output a latent feature representation $f_{G}$, as well as the parameters $\mu_{G}$ and $\text{log}\;\sigma_{G}^{2}$ for variational inference, where G∈{A,P} denotes two GCN branches.

The core graph convolution operation follows the standard GCN formulation:


(3) $$\text{GCN}(E,G)=\sigma \left({\tilde{D}}^{-\frac{1}{2}}\tilde{G}{\tilde{D}}^{-\frac{1}{2}}DW\right),$$

where G∈{A,P} is the graph adjacency matrix (with self-loops), $\tilde{D}=D+I$ is the diagonal degree matrix of $\tilde{G}$, **W** is a learnable weight matrix, and σ(·) is a non-linear activation function such as ReLU.

To model uncertainty and enable stochastic sampling in the latent space, we apply the reparameterization trick using the outputs $\mu_{G}$ and $\sigma_{G}$:


(4) $$z_{G}=\mu_{G}+\sigma_{G}⊙\epsilon ,\;\;\epsilon ∼N(0,I),$$

where ⊙ denotes element-wise multiplication. This process is applied independently to both the KNN and PPMI branches, resulting in two distinct sets of latent representations $z_{A}$ and $z_{P}$.

To fuse the complementary spatial features, we employ an attention-based graph fusion module that learns spot-specific attention weights $\alpha_{f},\alpha_{z}\in [0,1]$ to balance local and global contributions:


(5) $$\begin{array}{cc} & f=\alpha_{f}\cdot f_{A}+(1-\alpha_{f})\cdot f_{P},\\ & z=\alpha_{z}\cdot z_{A}+(1-\alpha_{z})\cdot z_{P}. \end{array}$$

Here, the parameter $\alpha_{f}$ and $\alpha_{z}$ are *learnable attention weights* that determines the relative contribution of local and global features for each spot, inspired by the Graph Attention Network (GAT) (Veličković *et al.* 2018). Specifically, given the two input feature vectors $f_{A}$ and $f_{P}$, the module computes the attention weight $\alpha_{f}$ through a linear transformation followed by a softmax normalization:


(6) $$\alpha_{f}=\frac{\;\text{exp}(w_{f}^{\prime }f_{A}+b_{f})}{\sum_{G\in \{A,P\}}\;\text{exp}\;(w_{f}^{\prime }f_{G}+b_{f})},$$

where $w_{f}$ and $b_{f}$ are learnable parameters. Similarly, the attention weight $\alpha_{z}$ for the latent representations $z_{A}$ and $z_{P}$ is computed as


(7) $$\alpha_{z}=\frac{\;\text{exp}(w_{z}^{\prime }z_{A}+b_{z})}{\sum_{G\in \{A,P\}}\;\text{exp}\;(w_{z}^{\prime }z_{G}+b_{z})}.$$

where $w_{z}$ and $b_{z}$ are also learnable parameters. This attention mechanism enables the model to adaptively balance information from different graph structures in a data-driven manner.

By concatenating f and z, the resulting embedding h=[f,z] encodes both gene expression profiles and multi-scale spatial context in a unified latent space.

### 2.5 Cluster classifier

In the source domain, where ground-truth annotations are available, the goal of the cluster classifier is to map the learned embeddings to accurate cluster assignments. Specifically, the latent representation h is fed into a classification network consisting of a stack of residual blocks followed by a softmax layer.

Each residual block refines the feature representation through non-linear transformation while preserving gradient flow and identity information. Formally, a residual block takes the form:


(8) $$\begin{array}{cc} & \text{ResBlock}(h)=h+\sigma (\text{BN}(W\cdot h)), \end{array}$$

where **W** is a learnable weight matrix, BN denotes batch normalization, and σ is a non-linear activation function.

After several residual blocks, the final output is passed through a softmax layer to produce a probability distribution over *C* predefined clusters:


(9) $$\begin{array}{c} \hat{y}=\text{Softmax}(\text{ResDecoder}(h))\in R^{N\times C}, \end{array}$$

where $\hat{y}$ denotes the predicted cluster probabilities for each spatial spot.

To supervise this process, we use the ground-truth cluster labels **y** available in the source domain and compute the cross-entropy loss:


(10) $$\begin{array}{c} L_{\text{cls}}=-\frac{1}{N}\sum_{i=1}^{N}\sum_{c=1}^{C}y_{ij}\;\text{log}({\hat{y}}_{ic}), \end{array}$$

where $y_{ic}\in \{0,1\}$ is the one-hot encoded true label, and ${\hat{y}}_{ic}$ is the predicted probability for cluster *c* at spatial spot *i*.

### 2.6 Domain adaptation

To transfer clustering-relevant knowledge from the labeled source domain to the unlabeled target domain, STransfer incorporates a generative-adversarial domain adaptation strategy. The key objective is to align the feature distributions of the two domains in the latent space, enabling the cluster classifier trained on the source slice to generalize effectively to the target slice without requiring manual annotations.

A domain discriminator *D* is introduced to distinguish whether a latent representation h is from the source or target domain. Simultaneously, the target encoder is optimized adversarially to fool the discriminator, thereby promoting domain-invariant feature representations.

The domain discriminator is optimized by minimizing the binary cross-entropy loss:


(11) $$\begin{array}{c} L_{D}=-E_{h^{s}}[\text{log}\;D(h^{s})]-E_{h^{t}}[\text{log}(1-D(h^{t}))], \end{array}$$

where $h^{s}$ and $h^{t}$ are embeddings from the source and target domains, respectively.

Conversely, the target encoder is trained to minimize the reverse objective, encouraging it to produce embeddings that make domain classification difficult:


(12) $$\begin{array}{c} L_{G}=-E_{h^{t}}[\text{log}\;D(h^{t})], \end{array}$$

thus promoting alignment between the distributions $P(h^{s})$ and $P(h^{t})$.

The model is trained in an alternating manner:

**Step 1**: Update the domain discriminator *D* to minimize $L_{D}$.

**Step 2**: Update the target encoder to minimize $L_{G}$.

This adversarial setup enables STransfer to learn domain-invariant yet cluster-discriminative embeddings, facilitating effective knowledge transfer from annotated slices to adjacent unlabeled ones.

## 3 Results

To evaluate the performance of STransfer, we conducted extensive experiments on five publicly available spatial transcriptomics datasets (DLPFC, HPR, mPFC, CRC, and OC), spanning both human and mouse tissues. These datasets encompass diverse species, tissue types, disease contexts, and sequencing platforms, thereby providing a comprehensive benchmark for assessing the generalization ability and robustness of spatial domain adaptation methods.

### 3.1 Human dorsolateral prefrontal cortex

**Figure 2:**
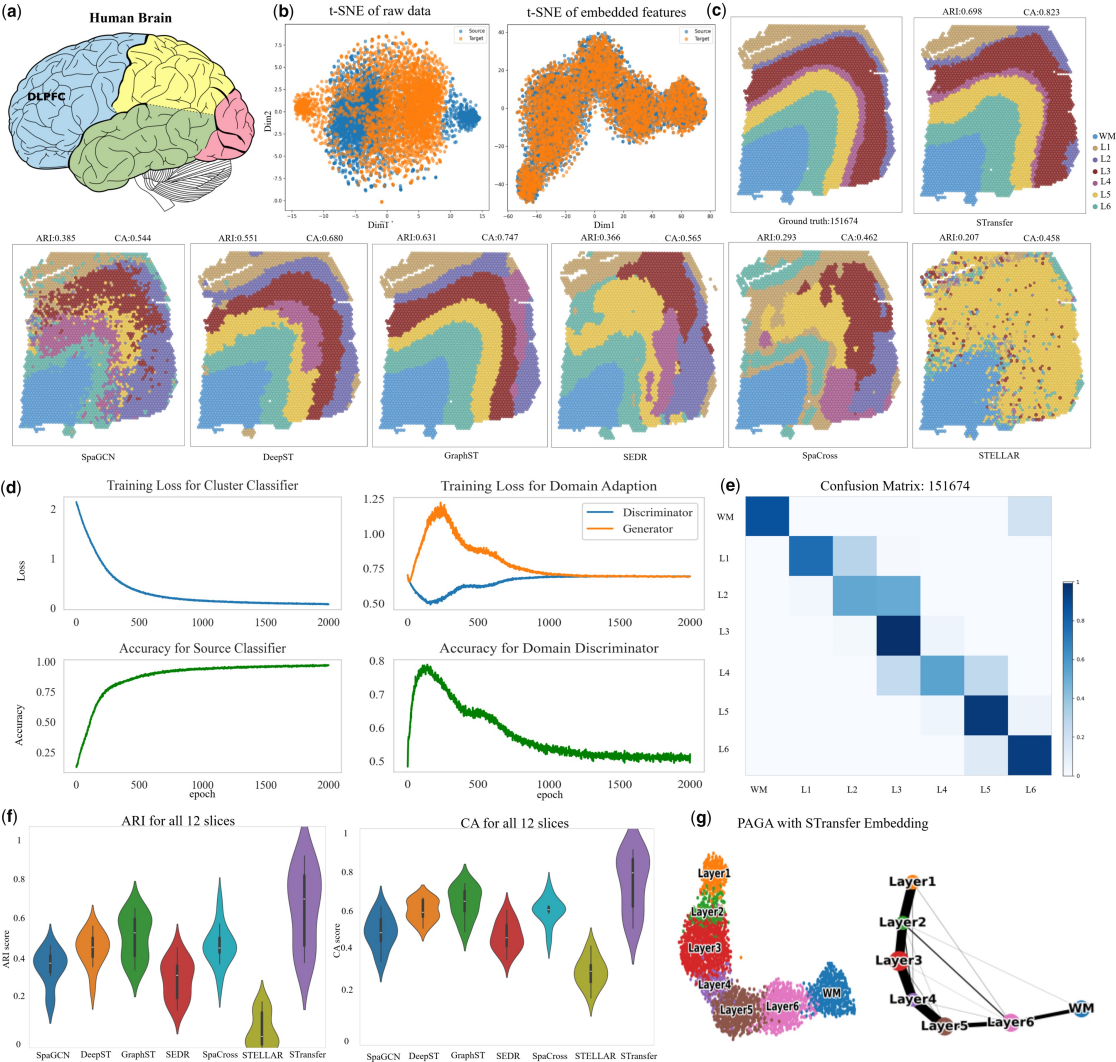
Validation on the DLPFC dataset. (a) Location of the dorsolateral prefrontal cortex in the human brain. (b) t-SNE plots of the raw gene expression data and the embedded features for both source domain (151 674) and target domain (151 673). (c) Visualization of clustering results for slice 151 673 predicted by various methods. (d) The training process of STransfer, including the variation of loss functions and classification accuracy over time. (e) Confusion matrix to illustrate the clustering and annotation performance of STransfer. (f) Violin plots summarizing the overall performance across all 12 tissue slices in the DLPFC dataset. (g) PAGA trajectory inference with STransfer embeddings.

We first evaluated the spatial clustering performance of STransfer on the human dorsolateral prefrontal cortex (DLPFC) dataset (Fig. 2a). The experimental design of DLPFC included two pairs of spatial replicates derived from three independent neurotypical adult donors. Each pair consisted of two directly adjacent serial tissue sections (10 μm apart), while the second pair was collected ∼300 μm posterior to the first, yielding a total of 12 samples processed using the 10x Genomics Visium platform. All sections were manually annotated into five to seven anatomically distinct regions, corresponding to cortical layers and white matter (WM).

To evaluate the domain adaptation capability of STransfer, we selected adjacent tissue sections from each spatial replicate pair, designating one as the source domain (with known manual annotations) and the other as the target domain (with labels withheld during model training). Using this setup, STransfer leverages the spatial and transcriptional knowledge from the source slice to guide clustering and annotation in the unlabeled target slice. The predicted annotations on the target domain were then quantitatively compared against its ground-truth manual labels using evaluation metrics such as adjusted Rand index (ARI) and clustering accuracy (CA). This design allows us to assess how well STransfer can transfer spatial structure and domain-invariant features across highly similar but distinct tissue sections.

We use the paired slices 151 673 and 151 674 from the DLPFC dataset as a representative example for visualization, where slice 151 674 serves as the source domain and slice 151 673 as the target domain. Figure 2b presents t-SNE visualizations of both the raw gene expression data and the embedded features. As shown, there exists a noticeable distributional shift between the source and target domains in the raw data. However, after feature embedding, the representations of the two domains become highly aligned and largely overlapping. This indicates that the embedding process effectively eliminates domain discrepancies, making it feasible to transfer label information from the source domain to the target domain via transfer learning.

Figure 2d illustrates the training dynamics of STransfer, including the variation of loss functions and classification accuracy over time. Using the labeled data from the source domain, the cluster classifier is trained in a supervised manner. The training loss of the cluster classifier steadily decreases and eventually converges, while the corresponding classification accuracy gradually increases, reaching ∼97%. During the domain adaptation process, the loss of the domain discriminator continuously decreases, while the negative loss of the generator increases accordingly. Eventually, both losses converge, and the domain classification accuracy stabilizes around 0.5. This indicates that the generator has successfully learned to produce domain-invariant features that can effectively fool the discriminator, validating the success of adversarial domain alignment.

Figure 2c visualizes the spatial organization of slice 151 673. The first panel shows the ground-truth anatomical annotations, while the remaining panels present the clustering results predicted by various methods, including our proposed STransfer model. As evidenced by the corresponding ARI and CA metrics, STransfer significantly outperforms existing approaches in recovering the underlying spatial domains, demonstrating superior alignment with the true anatomical structure.

Figure 2e presents the confusion matrix to illustrate the clustering and annotation performance of STransfer. All seven classes are well separated, as indicated by the strong diagonal blocks approaching a value of 1. Notably, unlike the other comparison methods, including SpaGCN, DeepST, GraphST, and SEDR, which are purely unsupervised and only provide clustering assignments without semantic labels, STransfer is capable of not only generating accurate clusters but also assigning them to the correct anatomical regions. This highlights STransfer’s advantage in interpretable and transferable spatial domain annotation.

Figure 2f illustrates a violin plot summarizing the overall performance across all 12 tissue slices in the DLPFC dataset. The results demonstrate that STransfer not only outperforms other methods on individual slices but also consistently achieves superior performance across the entire dataset. This highlights the robustness and generalizability of STransfer in spatial clustering and annotation tasks. For comparison, STELLAR (Brbić *et al.* 2022) is a supervised geometric deep learning method originally designed for single-cell–resolution spatial transcriptomics data. STELLAR relies on large numbers of individual cells to learn cell-type–specific geometric patterns and spatial relationships, which enables strong performance at the single-cell level. However, this design assumption limits its applicability to spot-based spatial transcriptomics data, where each spot typically aggregates multiple cells and the effective sample size is substantially smaller. As a result, STELLAR tends to exhibit reduced performance on conventional spot-level datasets, such as those generated by Visium.

Figure 2g illustrates the application of STransfer embeddings for trajectory inference. Specifically, we applied PAGA (Wolf *et al.* 2019) to the DLPFC slices. The resulting trajectories demonstrate that STransfer embeddings not only effectively distinguish distinct tissue states, but also preserve the underlying structural organization and continuous transition patterns between them, enabling biologically meaningful reconstruction of spatial and developmental relationships.

### 3.2 Mouse hypothalamic preoptic region

The second dataset analyzed originates from MERFISH technology and comprises five adjacent tissue sections obtained from the preoptic region of the mouse hypothalamus. This brain region plays a central role in regulating various social behaviors, such as reproduction and circadian rhythms, as well as essential homeostatic functions including neuroendocrine and cardiovascular regulation.

**Figure 3:**
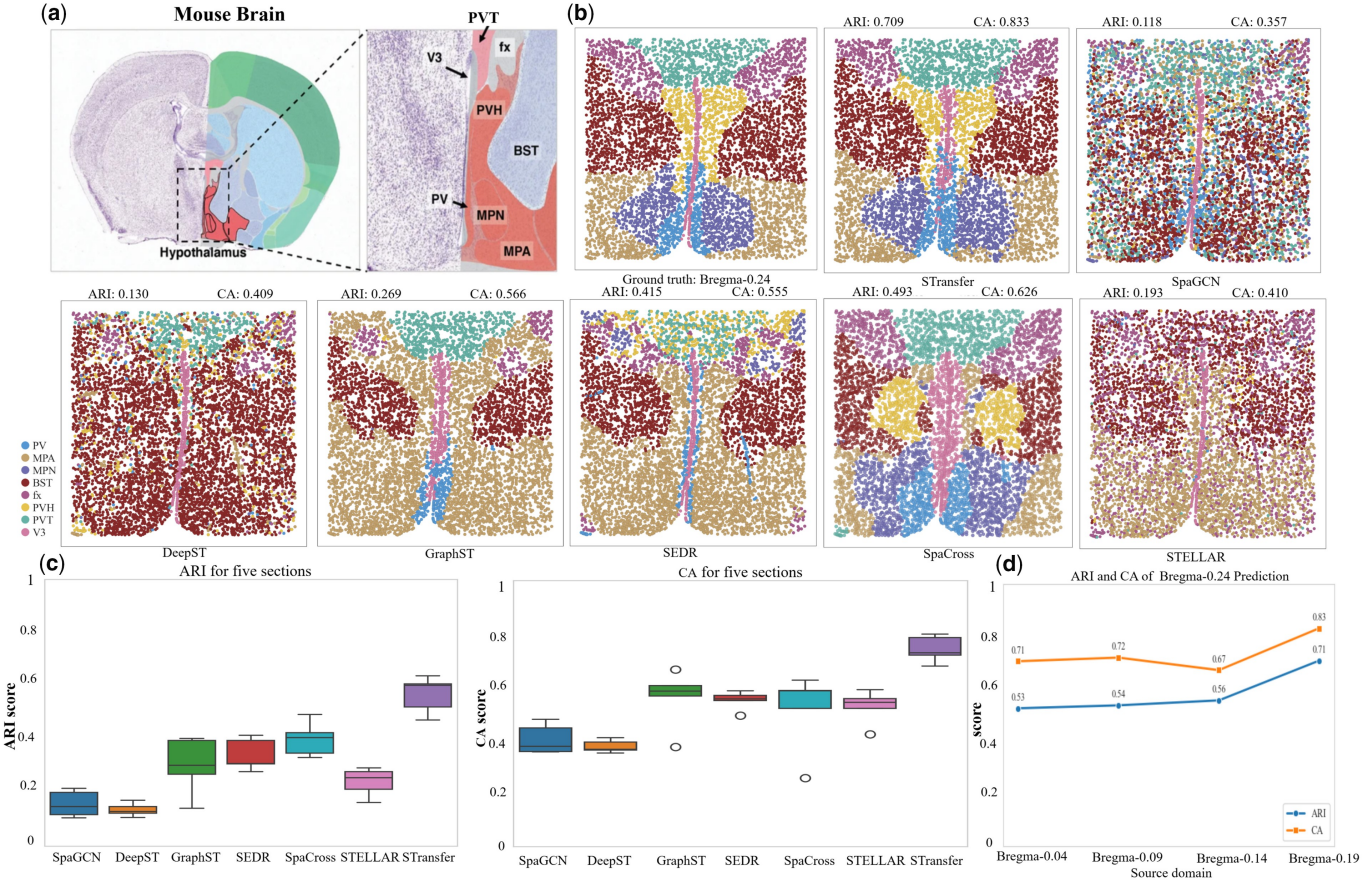
Validation on the HPR dataset. (a) The preoptic region of the mouse hypothalamus. (b) Visualization of clustering results for Bregma-0.24 predicted by various methods. (c) Boxplots summarizing the overall performance across four prediction tasks in the HPR dataset. (d) Prediction of Bregma-0.24 using different slices as source domains.

The five sections include Bregma-0.04 (5488 cells), Bregma-0.09 (5557 cells), Bregma-0.14 (5926 cells), Bregma-0.19 (5803 cells), and Bregma-0.24 (5543 cells), with gene expression profiled for a common set of 155 genes (Li and Zhou 2022). All cells across the sections were carefully annotated into eight anatomically distinct structures: the third ventricle (V3), bed nuclei of the stria terminalis (BST), columns of the fornix (fx), medial preoptic area (MPA), medial preoptic nucleus (MPN), periventricular hypothalamic nucleus (PV), paraventricular hypothalamic nucleus (PVH), and paraventricular nucleus of the thalamus (PVT) (Fig. 3a).

Since the five tissue sections in the HPR dataset are equally spaced, we applied the STransfer framework in a sequential transfer learning setting, where each preceding section was used as the source domain to predict the clustering and annotation of the immediately following section as the target domain. The training procedure followed the same protocol as described in Section 3.1.

Figure 3b visualizes the clustering results for the Bregma-0.24 slice as a representative example. The performance of STransfer is compared against several existing methods. The results demonstrate that STransfer consistently achieves superior performance in both ARI and CA, highlighting its effectiveness in leveraging spatial and cross-slice information for precise annotation.

Figure 3c presents a boxplot summarizing the overall performance of various methods across four prediction tasks: Bregma-0.04 → Bregma-0.09, Bregma-0.09 → Bregma-0.14, Bregma-0.14 → Bregma-0.19, and Bregma-0.19 → Bregma-0.24. The results clearly demonstrate that STransfer consistently outperforms other methods in terms of both ARI and CA, confirming its robustness and superior generalization ability in cross-slice spatial transcriptomics analysis.

Figure 3d shows the prediction results for Bregma-0.24 as the target domain, using different slices as source domains. The results indicate that closer source slices yield higher prediction accuracy, consistent with the biological similarity of adjacent sections. Notably, even the most distant slice still achieves favorable prediction performance, suggesting that STransfer exhibits strong long-range transferability and is capable of effectively leveraging information across spatially distant tissue sections.

### 3.3 Mouse medial prefrontal cortex

**Figure 4:**
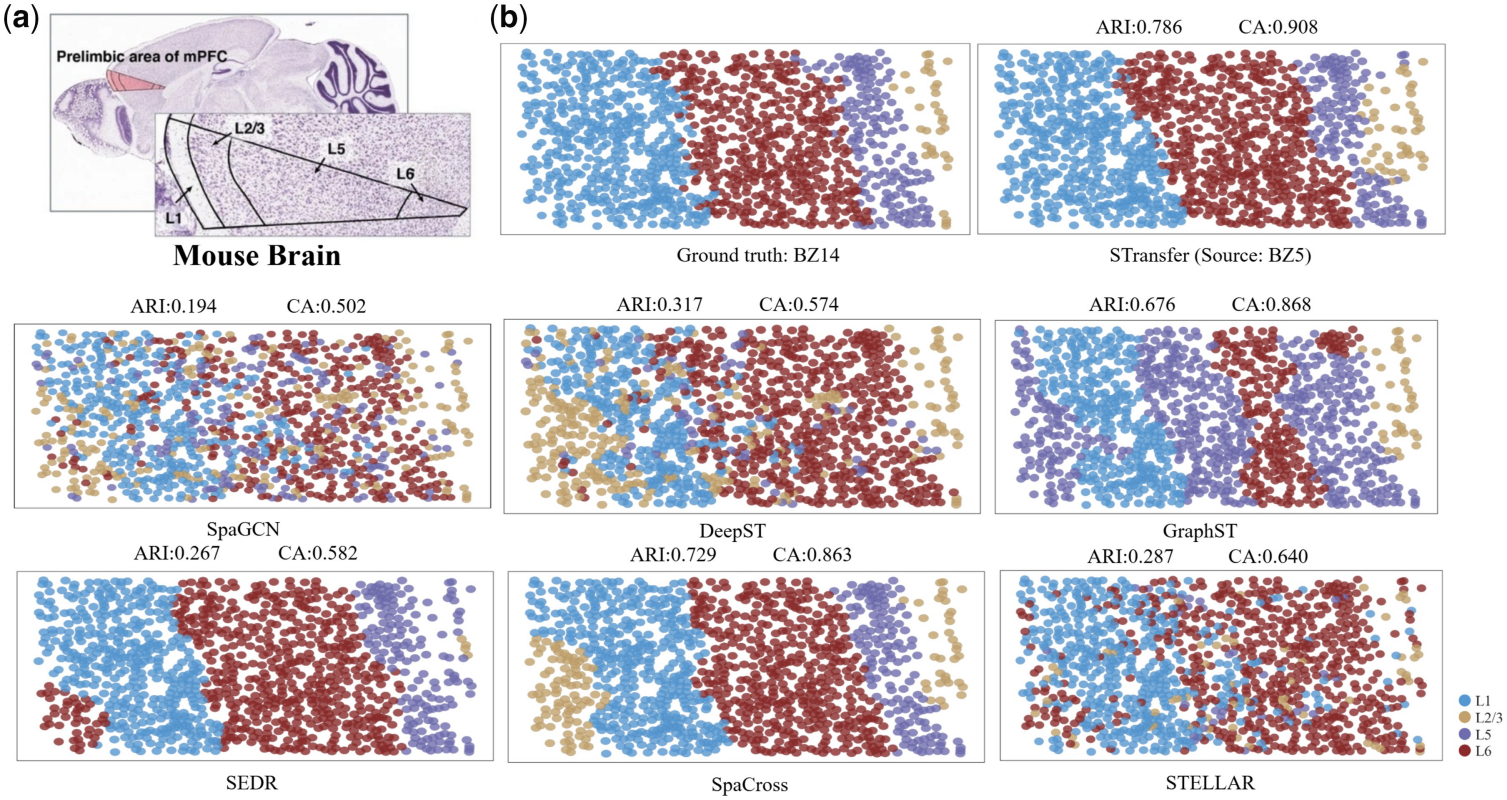
Validation on the mPFC dataset. (a) Cells within mPFC are categorized into four spatial domains corresponding to cortical layers L1, L2/3, L5, and L6. (b) A clustering example in which BZ14 is used as the target domain and BZ5 as the source domain.

This section evaluates the performance of STransfer on the mouse mPFC dataset, which comprises three annotated tissue slices–BZ5, BZ9, and BZ14–each obtained from the same mPFC region but collected from different individual mice. Cells within these slices are categorized into four spatial domains corresponding to cortical layers L1, L2/3, L5, and L6 (Fig. 4a), based on the spatial expression patterns of well-established marker genes, including Bgn (L1), Cux2 (L2/3), Tcerg1l (L5), and Pcp4 (L6) (Li and Zhou 2022). This experimental design enables the evaluation of STransfer’s cross-individual transfer learning ability.

Similar to the training procedure described in Section 3.1, STransfer performs pairwise transfer learning between the three slices. Specifically, each pair of slices is used bidirectionally–one as the source domain and the other as the target domain–allowing a comprehensive evaluation of cross-individual transfer performance. Tables 2 and 3 summarize the transfer results across different slice pairs, as well as comparisons with other existing methods. Both the ARI and CA demonstrate that STransfer not only achieves stable performance across different source domains but also significantly outperforms competing methods.

**Table 2:** ARI of various methods on the three BZ samples.

STransfer		Source ID			Competing methods					
		BZ5	BZ9	BZ14	SpaGCN	DeepST	GraphST	SEDR	SpaCross	STELLAR
**Target ID**	**BZ5**	–	0.794	0.625	0.286	0.387	0.459	0.835	0.714	0.243
	**BZ9**	0.735	–	0.706	0.240	0.393	0.562	0.598	0.314	0.250
	**BZ14**	0.785	0.708	–	0.194	0.316	0.675	0.267	0.729	0.153
**Average**		**0.726**			**0.240**	**0.365**	**0.565**	**0.567**	**0.586**	**0.215**

**Table 3:** Clustering accuracy of various methods on the three BZ samples.

STransfer		Source ID			Competing methods					
		BZ5	BZ9	BZ14	SpaGCN	DeepST	GraphST	SEDR	SpaCross	STELLAR
**Target ID**	**BZ5**	–	0.917	0.816	0.585	0.642	0.689	0.938	0.896	0.630
	**BZ9**	0.898	–	0.860	0.544	0.663	0.813	0.814	0.625	0.640
	**BZ14**	0.908	0.848	–	0.501	0.574	0.867	0.581	0.863	0.565
**Average**		**0.875**			**0.543**	**0.626**	**0.790**	**0.778**	**0.795**	**0.612**

Figure 4b visualizes an example in which BZ14 is used as the target domain and BZ5 as the source domain. The results demonstrate that STransfer is capable of accurately classifying and annotating spatial regions, closely aligning with the ground-truth annotations.

### 3.4 Human colorectal cancer tumors

**Figure 5:**
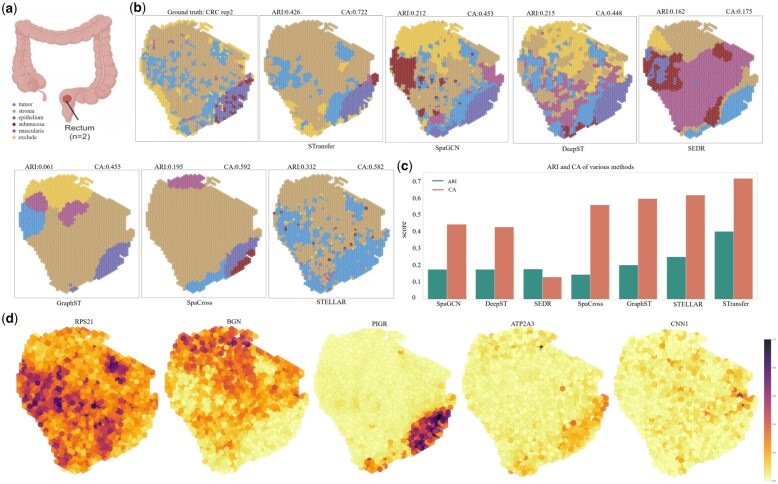
Validation on the CRC dataset. (a) Anatomical localization of the CRC tissue sections generated using the 10x Genomics Visium platform. (b) UMAP visualization of the second replicate, showing the ground-truth annotations alongside the clustering and annotation results produced by STransfer and other competing methods. (c) Bar plot comparing the average ARI and CA scores across different methods. (d) Spatial expression patterns of representative marker genes.

To further validate the effectiveness of STransfer on disease-related data, we conducted additional experiments on a colorectal cancer (CRC) dataset (Valdeolivas *et al.* 2024). This dataset comprises a pair of serial tissue sections from a CRC tumor patient, generated as technical replicates using the 10x Genomics Visium platform on fresh-frozen resection samples. The two sections contain 1691 and 2128 Visium spots, respectively, with each spot capturing a total of 36 601 genes. Figure 5a outlines the anatomical localization of the CRC samples.

Tumor tissues typically exhibit more dispersed and heterogeneous spatial patterns than normal tissues; therefore, strong classification performance in tumor samples indicates the robustness and reliability of the proposed model. In this study, all spots were grouped into six major tissue domains: tumor, stroma, epithelium, submucosa, muscularis or immune-cell aggregates, and a final category of low-quality regions annotated as excluded regions.

We treated the first replicate as the source domain and the second replicate as the target domain. Figure 5b shows the ground-truth annotations of the second replicate under UMAP dimensionality reduction, together with the predictions generated by STransfer and those from other competing models. Figure 5c further reports the average ARI and CA scores obtained when the two replicates are alternately used as the source and target domains. The results demonstrate that STransfer consistently outperforms all baseline methods, achieving significantly higher performance in terms of both ARI and CA.

In addition, Fig. 5d visualizes the expression levels of several representative marker genes for each tissue region, including RPS21, BGN, PIGR, ATP2A3, and CNN1. These expression patterns are highly consistent with known biological annotations, further confirming that the clustering results produced by STransfer closely match the ground truth.

### 3.5 Ovarian cancer datasets across multiple platforms

To further assess the robustness and effectiveness of STransfer, we conducted additional evaluations on an ovarian cancer (OC) dataset (Ren *et al.* 2025). This dataset comprises multiple spatial transcriptomics tissue sections obtained from the same OC patient and profiled across four high-throughput platforms with subcellular resolution: Stereo-seq, Visium HD, CosMx, and Xenium. By capturing the same biological system using distinct sequencing technologies, this dataset provides a challenging and realistic benchmark for cross-platform spatial domain adaptation.

**Figure 6:**
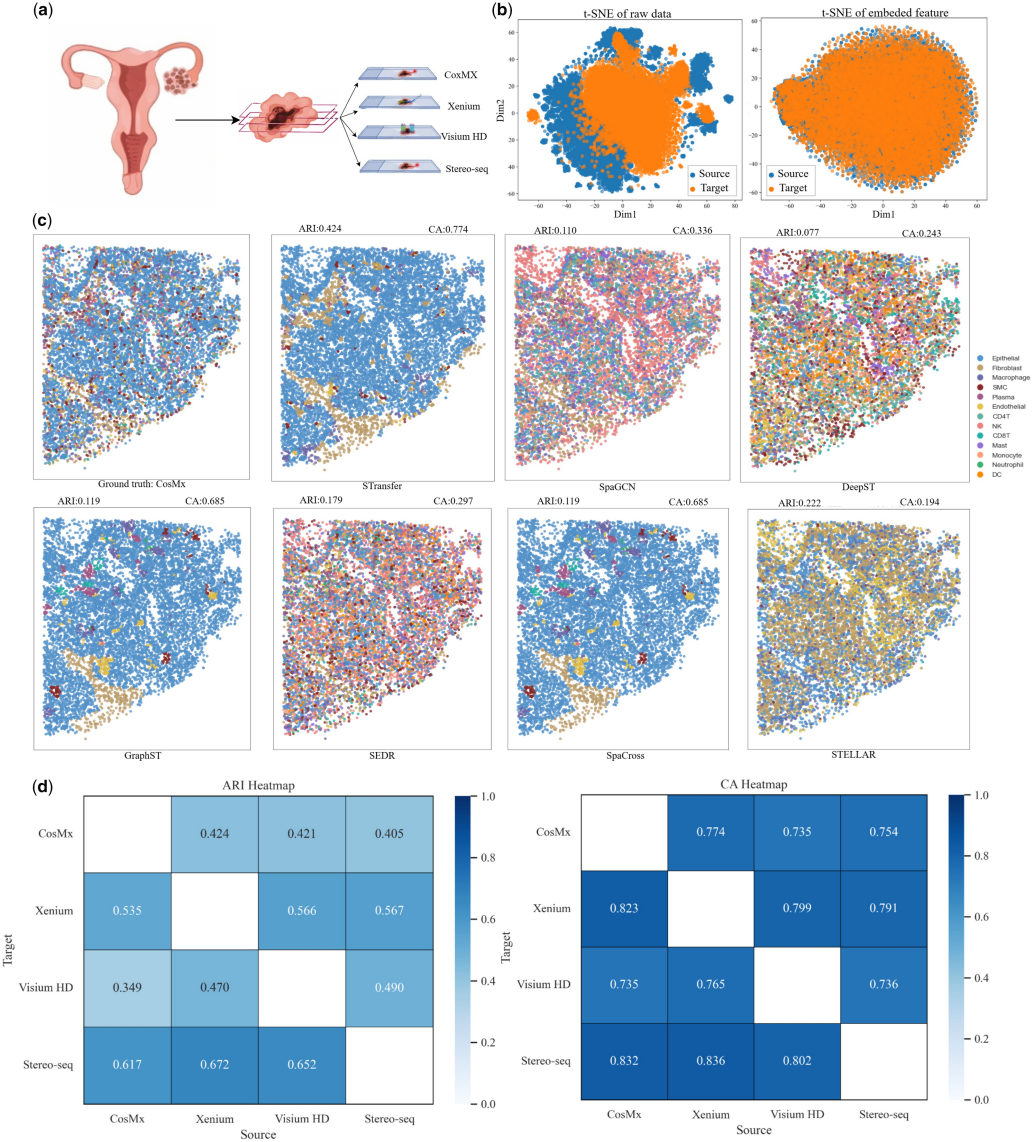
Validation on the OC Dataset. (a) Anatomical structure of the OC tissue sections and the cross-platform relationships among multiple slices profiled using different spatial transcriptomics technologies. (b) t-SNE visualizations of raw gene expression data and learned embeddings for a representative cross-platform transfer setting (Xenium as source and CosMx as target). (c) UMAP visualizations comparing STransfer predictions on the target domain with ground-truth annotations and results from competing methods. (d) Heatmap of ARI and CA scores for pairwise cross-platform transfers among the four tissue slices.

Each tissue section was manually annotated by domain experts into 13 tumor–microenvironment regions, enabling a consistent and reliable reference for quantitative evaluation. The substantial differences in gene coverage, spatial resolution, and measurement characteristics across platforms introduce pronounced technical heterogeneity, making accurate knowledge transfer across domains particularly difficult. Evaluating STransfer on this dataset therefore allows us to rigorously examine its ability to learn platform-invariant spatial representations while preserving biologically meaningful structures across diverse spatial transcriptomics technologies. Figure 6a illustrates the anatomical organization of the ovarian cancer tissue sections, as well as the relationships among multiple slices profiled across different sequencing platforms.

For the spatial transcriptomics slices generated by the four different sequencing platforms, we paired them in a pairwise manner, alternately designating one as the source domain and the other as the target domain, to systematically evaluate the cross-platform transfer capability of STransfer. Figure 6b and c presents a representative example in which the Xenium slice is used as the source domain and the CosMx slice as the target domain.

Specifically, Fig. 6b shows the t-SNE visualizations of both the raw gene expression data and the learned embedding features for the source and target domains. The results clearly demonstrate that, after transfer learning, the embedding process effectively mitigates platform-induced domain discrepancies, aligning samples from different technologies into a shared feature space. This alignment enables reliable transfer of label information from the source domain to the target domain, highlighting the effectiveness of STransfer in cross-platform spatial domain adaptation.

Figure 6c presents the UMAP visualization of the target domain under STransfer and compares its predicted labels with the ground truth as well as the results from other competing models. The results demonstrate that, even in a challenging multi-class setting with 13 tissue regions, STransfer achieves substantially higher clustering accuracy, significantly outperforming alternative methods in terms of both ARI and CA.

Figure 6d shows a heatmap summarizing the prediction performance for pairwise cross-platform transfers among the four tissue slices. The ARI and CA results indicate that STransfer exhibits strong cross-platform stability and consistency, demonstrating its robustness when transferring knowledge across different spatial transcriptomics technologies.

### 3.6 Ablation study

In this section, we conduct a comprehensive ablation study on the DLPFC dataset to systematically analyze the contribution of each key component in the STransfer framework. Specifically, we focus on three core modules: dual-graph convolution, cluster classifier, and the domain adaptation module. By selectively removing or modifying these components and comparing the resulting performance, we aim to elucidate their individual roles and overall importance within the model.

The dual-graph convolution module integrates complementary spatial information by jointly modeling local spatial adjacency and long-range structural dependencies, allowing the model to capture both fine-grained neighborhood interactions and global tissue organization. In contrast, relying on a single graph representation—using either KNN or PPMI alone—leads to a noticeable decline in clustering accuracy, highlighting the importance of multi-scale spatial modeling for robust spatial domain identification.

The cluster classifier plays a central role in guiding discriminative representation learning by explicitly enforcing cluster-level supervision during training. When this component is removed or replaced with a simple multi-layer perceptron (MLP), the model lacks a direct mechanism to align the learned embeddings with biologically meaningful tissue domains, resulting in less coherent clusters and reduced annotation accuracy.

The domain adaptation module is designed to mitigate distribution shifts between source and target domains, which commonly arise from batch effects or differences in experimental conditions. When this module is ablated, performance drops significantly, particularly in cross-slice and cross-domain settings, demonstrating its essential role in learning domain-invariant representations and enabling effective knowledge transfer.

Overall, the ablation results on the DLPFC dataset confirm that each component of STransfer contributes substantially to its performance, and that the synergy among dual-graph convolution, cluster classification, and domain adaptation is critical for achieving robust and accurate spatial domain adaptation. Table 4 summarizes the average ARI and clustering accuracy across all 12 DLPFC slices under different module combinations. The results clearly demonstrate that the full STransfer model achieves the best performance among all ablated variants.

**Table 4:** Ablation study on the DLPFC dataset.

Models	KNN	PPMI	Classifier	MLP	Adaptation	ARI	CA
Baseline	√	√	√	×	√	0.637	0.749
1	√	×	√	×	√	0.581	0.706
2	×	√	√	×	√	0.625	0.747
3	√	√	×	√	√	0.562	0.681
4	√	√	√	×	×	0.132	0.441

## 4 Conclusion

In this study, we proposed STransfer, a transfer learning framework designed to improve spatial transcriptomics analysis by leveraging annotated reference slices to infer spatial domains in unannotated target slices. STransfer integrates three core components: a feature encoder for robust representation learning, a cluster classifier trained using source domain labels, and an adversarial domain discriminator to align feature distributions between source and target domains. By jointly optimizing the classification loss and domain adaptation loss, STransfer effectively learns domain-invariant features that enable accurate and interpretable clustering across diverse spatial contexts.

We systematically evaluated the performance of STransfer across three benchmark ST datasets. On the DLPFC dataset, visualization and quantitative metrics (ARI and CA) demonstrated that STransfer effectively bridges domain shifts between adjacent human brain slices. On the HPR dataset, which consists of sequential mouse brain sections with uniform spacing, STransfer showed strong capability in both short-range and long-range spatial transfer, achieving robust performance across multiple slice-pair combinations. Furthermore, experiments on the mouse mPFC dataset confirmed the model’s ability to generalize across individual animals, highlighting its effectiveness in cross-subject transfer scenarios.

In addition, we evaluated STransfer on a CRC dataset to assess its performance on disease-related tissues, which typically exhibit higher spatial heterogeneity and more complex microenvironmental structures. The strong performance on CRC samples demonstrates the reliability of STransfer in challenging pathological settings. We further validated the model on an OC dataset profiled across multiple spatial transcriptomics platforms and multiple tissue slices from the same patient. This cross-platform evaluation highlights STransfer’s robustness to platform-specific technical variations and its ability to learn platform-invariant spatial representations. Collectively, these results demonstrate the strong robustness and generalization capability of STransfer across diverse biological conditions, disease states, and sequencing technologies.

Despite its strong performance, STransfer has several limitations. It relies on at least one well-annotated slice as a source domain, which may limit its applicability in cases where labeled data is scarce or unavailable. Additionally, while it performs well in pairwise or small-scale transfer settings, scaling to large, heterogeneous cohorts (e.g. across developmental stages or disease states) may require further methodological enhancements.

Looking forward, future research could explore semi-supervised and self-supervised variants of STransfer to relax the requirement for annotated source domains. We also plan to investigate multi-source transfer learning strategies, where knowledge from multiple annotated slices is integrated to improve robustness and accuracy across diverse target domains.

In conclusion, STransfer provides a unified, flexible, and accurate solution for spatial domain annotation in ST datasets. By enabling effective knowledge transfer and preserving biological interpretability, it addresses key limitations of unsupervised methods and opens new avenues for scalable, data-efficient spatial omics analysis.

## Data Availability

All datasets used in our study were obtained from previously published sources. The DLPFC dataset is publicly available through the spatialLIBD project. The HPR and mPFC datasets correspond to Data 25–29 and Data 31–33, respectively, and can be accessed from the SDMBench repository. The CRC dataset is available for download from the CRC website. The OC dataset can be obtained from the Spatch project.

## Code availability

The code for STransfer has been uploaded to GitHub: https://github.com/Saki-JSU/Publications/tree/main/STransfer.
